# Signaling in colon cancer stem cells

**DOI:** 10.1186/1750-2187-7-11

**Published:** 2012-08-06

**Authors:** Sanchita Roy, Adhip PN Majumdar

**Affiliations:** 1 John D Dingell VA Medical Centre, 4646 John R; Room: B-4238, Detroit, MI, 48201, USA; 2 Department of Internal Medicine, Wayne State University School of Medicine, Detroit, MI, 48201, USA; 3 Karmanos Cancer Centre, Wayne State University School of Medicine, Detroit, MI, 48201, USA

**Keywords:** Colon cancer, Cancer stem cell, Notch, Wnt, TGF-β, Hedgehog

## Abstract

**Abstract:**

Colorectal cancer is the fourth most common form of cancer worldwide and ranks third among the cancer-related deaths in the US and other Western countries. It occurs with equal frequency in men and women, constituting 10% of new cancer cases in men and 11% in women. Despite recent advancement in therapeutics, the survival rates from metastatic are less than 5%. Growing evidence supports the contention that epithelial cancers including colorectal cancer, the incidence of which increases with aging, are diseases driven by the pluripotent, self-renewing cancer stem cells (CSCs). Dysregulation of Wnt, Notch, Hedgehog and/or TGF-β signaling pathways that are involved in proliferation and maintenance of CSCs leads to the development of CRC. This review focuses on the signaling pathways relevant for CRC to understand the mechanisms leading to tumor progression and therapy resistance, which may help in the development of therapeutic strategies for CRC.

## 

Colorectal cancer, the third most common cancer in women and the fourth in men, causes 610,000 deaths per year worldwide [1]. The American Cancer Society has reported 101,340 new cases of colon and 39,870 cases rectal cancers and 49,380 deaths in 2011 in the US. The primary cause of death is due to liver metastasis [2]. Despite recent advances in medicine nearly 50% of patients with colorectal cancer show tumor recurrence. Although the reason for this is not fully understood, the presence of chemotherapy-resistant cancer stem (CSCs) is thought to be one the primary causes for tumor recurrence [3]. A number of signaling pathways, most notably Wnt/β-catenin, notch and Hedgehog play important roles in maintaining the growth and functional integrity of CSC. Therefore, a better understanding of the signaling mechanisms in CSC will aid in the development of new therapeutic strategies for CRC.

## Stem cell niche in the colon

The colon or large intestine consists of five layers: - the mucosa, submucosa, muscularis mucosa, muscularis externa, and serosa. The flat mucosal surface is lined by an absorptive and secretory epithelium (simple columnar) interspersed with deep crypts projecting deep into the underlying submucosa called crypts of Lieberkuhn. Normal human colon consists of millions of crypts, each containing about 2000 cells. Three main epithelial cell lineages comprise a crypt: the columnar cells or colonocytes, the mucin-secreting cells or goblet cells, and the enteroendocrine cells. A fourth category Paneth cell is occasionally present in ascending colon or in certain disease condition [4]. They play important roles in host defense to protect stem cells. The adult mammalian intestinal epithelium is unique for its rapidly self renewing property. Under normal circumstances, turnover of epithelial cell lineages occurs constantly in every 2–7 days but this process become faster during tissue repair and wound healing [5]. The regeneration is maintained by adult stem cells. According to unitarian theory, proposed in 70s, all four cell lineages within the gastrointestinal epithelium are clonal populations derived from a slowly cycling clonogenic stem cells (SC) [6]. The upper one-third of the colonic crypt consists of differentiated cells (colonocytes, goblet cells, enteroendocrine cells) and lower two-third is predominantly composed of transit-amplifying cells (TA cells). Stem cells are located at the bottom of the crypt and 5-10 stem cells are predicted to be present in each crypt [7]. At the crypt base, in the vicinity of basal lamina, stem cells are surrounded by mesenchymal cells that are of myofibroblast lineage. These pericryptal myofibroblasts produce the Wnt signalling ligands which bind to Frizzled receptors as well as morphogenetic protein (BMP) antagonists gremlin 1(GREM1), gremlin 2 (GREM2) on basal epithelial stem cells and also can modulate notch signaling. They can regulate stem cell functions by modulating effects of Wnt signalling on ephrin B1 (EFNB1) and its receptors EPHB2, EPHB3 in the stem cells [4].

## Colon cancer stem cells

German pathologist Rudolf Virchow was the first to propose CSC hypothesis in 1855. He predicted that cancers occur upon activation of dormant embryonic-like cancerous cells present in mature tissues but not spontaneously [8]. It was Lapidot who, subsequently, proved the CSC hypothesis in 1994. He demonstrated that human acute myeloid lymphoma cells, bearing the stem cell characters, produced leukemia in immune compromised mice [9]. After this discovery, the presence of CSCs was searched in solid tumors [10]. In 2007 O'Brien and Vitiani, individually, discovered colon cancer stem cells. O'Brien *et al* purified CD133^+^ human colon cancer-initiating cell (CC-IC) and transplanted them into renal capsule of immunodeficient mice. Using limiting dilution analysis they showed 1/5.73 × 10^4^ unfractionated tumor cells produced tumor in immunodeficient mice while 1/262 of CC-IC in CD133^+^ cells formed tumor [11]. Vitiani *et al* reported that only 2.5% of the tumorigenic cells in colon cancer had high CD133 expression. Subcutaneous injection of CD133^+^ colon cancer cells produced tumors in immunodeficient mice, whereas CD133^-^ cells did not. Serial transplantation of such tumor in several generations gives rise to tumor with identical phenotype. These cells can also grow in serum free media *in vitro* bearing the same antigenic character and transplantation ability like the original tumor [12].

In terms of self-renewal and multipotency for differentiation into a particular type, CSCs are very much similar to normal adult stem cells. Because of their scarcity among tumor mass, identification and characterization of CSC remain a technical challenge. However, putative stem cell markers are being used to isolate CSCs. Discussion on stem cell markers is not a scope of this article, but for the sake of convenience markers of normal and colon cancer stem cells are listed Table 1.

**Table 1:** **Markers to identify colonic SCs and colon CSCs **[13]

	**Markers**	**Cellular Functions**
Normal Stem Cell	Musahi-1	RNA binding protein
	Hes-1	Transcriptional repressor
	EphB receptors	Cell surface receptors
	Bmi-1	Policomb-repressor protein
	Lgr-5	Wnt target gene
	ALDH1A1	Enzyme
	DCAMKL1	Kinase
Colon Cancer Stem Cell	CD133	Associated with poor prognosis, low survival, and distant metastasis in colorectal adenocarcinoma.
	CD44	Hyaluronic acid receptor
	CD166	Call adhesion molecule
	ALDH1A1	Enzyme
	ATP binding cassette protein	Drug transporter, effluxes drugs.
	OCT4	POU-domain transcription factor, highly expressed in embryonic stem (ES) cells.
	SOX2	Group B of the Sox family of transcription factor, involved in development.
	c-Myc	Transcription factor, high c-Myc levels blocks cell differentiation and enhance self-renewal of committed and differentiated cells
	B-Integrin (CD29)	Cell adhesion molecule

To date, therapeutic strategies in the treatment of solid tumors have been focused on targeting specific cellular signaling pathway that regulates cell proliferation, apoptosis, and angiogenesis. In the present communication, we will briefly describe the Wnt/β-catenin, TGF-β, Notch and Hedgehog signaling pathways that are believed to regulate CSC functions.

## Wnt signaling

Wnt signaling pathway plays a pivotal role in the regulation of epithelial stem cell self renewal [14,15]. In contrast, dysregulation of this signaling has been implicated in many epithelial cancers, including colon carcinogenesis [16,17]. Wnt signals are either transduced to the canonical Wnt pathway for cell fate determination or to the non-canonical Wnt pathway for controlling the tissue polarity and cell movement. Canonical and non-canonical Wnt pathways are activated by different Wnt ligands for example Wnt1, 2, 3, 3A, 8A, 8B, 10A, 10B are known to induce canonical Wnt pathway, whereas Wnt4, 5A, 5B, 6, 7A, and 7B are needed for non-canonical Wnt pathway [18,19]. Canonical Wnt signals are transduced through Frizzled/ LRP5/6 complex to stabilize β-catenin by inhibiting its phosphorylation-dependent degradation and to activate downstream target TCF/LEF to regulate specific gene expression. The two best-studied Wnt non-canonical Wnt pathways are the Planar Cell Polarity (PCP) and Wnt/Calcium Pathways [20].

The function of Wnt signaling in adult tissue homeostasis is best studied in the gut, where a gradient of Wnt signaling activity is required for the compartmentalization. Wnt signaling components are present throughout the crypt [21]; while active canonical signals are critical to maintain stem cells located at the bottom of the crypt. Blockade of Wnt signaling, either by artificial deletion of TCF4 or overexpression of the Wnt antagonist Dickkopf-1 (DKK1), results in loss of epithelial cell proliferation and intestinal tissue structure [22].

Mutation induced inactivation of the APC gene or activating mutations of β-catenin is reported in all CRC patients [23] which are also the major cause of malignant transformation [24]. The loss of function of Wnt signaling components are responsible for the pathogenesis of CRC [25]. Mutations result in stabilization of β-catenin and everlasting activation of the Wnt-induced transcription of a number of cell survival genes, even in the absence of any extracellular signals.

In spite of the mutations in APC or β-catenin genes leading to constitutive activation of the Wnt signalling cascade, most colorectal cancers show cellular heterogeneity of β-catenin indicating a more complex regulation of Wnt signalling. When this heterogeneity was analysed with a Wnt reporter construct, it is observed that high Wnt activity functionally designates the colon cancer stem cell (CSC) population. In adenocarcinomas, high activity of the Wnt pathway is found in tumor cells located close to stromal myofibroblasts, signifying that Wnt activity and cancer stemness could be regulated by extrinsic signals. For example, myofibroblast-secreted hepatocyte growth factor activates β-catenin-dependent transcription and subsequently CSC clonogenicity. Moreover, myofibroblast-secreted factors also restore the CSC phenotype in more differentiated tumor cells both *in vitro* and *in vivo*[26].

The non-canonical Wnt signaling is reported to antagonize β-catenin dependent transcription [27], suggesting an anti-oncogenic effect of non-canonical Wnt signaling. However, VANGL1, a PCP pathway protein, has been shown to promote metastasis of colon cancer.

Wnt/β-catenin pathway can also regulate the growth and maintenance of colonospheres, which are considered to be surrogate tumors. Colon CSCs can form floating spheroids under anchorage-independent conditions in a serum-free defined media. Studies from this laboratory have demonstrated that, while colonospheres have reduced membrane bound β-catenin, they exhibit increased levels of total β-catenin, cyclin-D1 and c-myc and down regulation of axin-1 and phosphorylated β-catenin [28]. Increased expression of β-catenin is associated with induction of transcriptional activation of TCF/LEF, which is decreased when β-catenin is silenced using siRNA [28], This leads to decreased colonosphere formation [28]. In contrast, upregulation of c-myc, a down-stream effector of TCF/LEF, greatly enhances the formation of colonospheres [28].

## TGF-β signaling

The TGF-β signaling pathway is one of the most commonly altered pathways in human cancers [29]. This pathway regulates cell proliferation, differentiation, migration, apoptosis, stem cell maintenance and function. TGFβ superfamily ligands bind to a type II serine/threonine kinase receptor, which recruits and phosphorylates type I receptor. The type I receptor in turn phosphorylates receptor-regulated SMADs (R-SMADs) which can bind the comediator Smads (coSMAD). R-SMAD/coSMAD complexes accumulate in the nucleus where they act as transcription factors and participate in the regulation of target gene expression. To date, eight Smad proteins have been identified and classified into three functional classes- (i) receptor-activated Smads (R-Smads): Smad1, Smad2, Smad3, Smad5, Smad8; (ii) comediator Smads (coSMAD): Smad4, Smad10; (iii) inhibitory Smads: Smad6, Smad7. Smad proteins function through adaptor proteins such as SARA and β2SP and by interacting with multiple other signal transduction pathways. Downstream targets of TGF-β signaling are key cell-cyclecheckpoint genes including CDKN1A (p21), CDKN1B (p27), and CDKN2B (p15) [30].

In normal intestinal epithelium the tumor suppressor function of TGF-β includes inhibition of cell proliferation and induction of apoptosis. However, in many CRCs escape the tumor-suppressor effects of TGF- β, hence become resistant to TGFβ-induced growth inhibition [31]. Interestingly TGF- β can switch its own role from a tumor suppressor to a tumor promoter. TGFβ-induced proliferative pathways are activated if SMAD signaling is abrogated. It has been demonstrated that TGFβ activates PI3K to downregulate *PTEN* for enhancement of cell proliferation that is independent of SMAD proteins [32]. Nearly 80% of CRCs is associated with frameshift mutations of TBR2 which is an outcome of errors prone replication of microsatellite sequences present in TBR2 gene [33]. Mutations in the type I receptor (TBR1), Smad2, Smad4 have been reported for CRC [34].

Loss of β2SP in combination with loss of Smad4 is found in advanced and metastatic CRC [35].

## Role of Notch signaling in normal and cancerous colon

Notch signaling, an evolutionarily conserved pathway in multicellular organisms, regulates cell-fate determination during development and in stem cells. It mediates juxtacrine signaling among adjacent cells. Notch receptors are single-pass trans-membrane proteins composed of functional extracellular (NECD), transmembrane (TM), and intracellular domains. Interaction between Notch and its ligands initiates a signaling cascade that regulates differentiation, proliferation, and apoptosis. The core elements of the Notch signaling system are the Notch receptor, DSL ligands (Delta and Serrate/Jagged in Drosophila and vertebrates, Lag2 in *C. elegans*) and CSL DNA-binding proteins (CBF1/RBPJ-κ in vertebrates, Su(H) [Suppressor of hairless] in Drosophila, Lag1 in *C. elegans*). Four paralogs of the Notch gene- Notch1, 2, 3 and 4, and five Notch ligands- Jagged1, Jagged2, Delta1, Delta2 and Delta3, have been identified in vertebrates [36]. Notch proteins contain extracellular EGF (Epidermal Growth Factor)-like repeats, which interact with the DSL domain of ligands. Activation of Notch upon ligand binding is followed by proteolytic cleavage releasing an intracellular domain of Notch (NICD) from the membrane tether. The NICD contains the RAM23 domain (RAM), which enhances interaction with CSL protein; NLS (Nuclear Localization Signals); a CDC10/Ankyrin repeat domain ANK, which mediates interactions with CSL and other proteins, and a PEST domain rich in proline, glutamate, serine and threonine residues [37]. Next the Notch COOH-terminal fragment is cleaved by γ-secretase (includes Presenilin and Nicastrin) to release NICD into the cytoplasm. Upon release, the NICD translocates to the nucleus and associates with the CSL [CBF1/RBPJ-κ/ in vertebrates, Su (H) in Drosophila, and Lag-1 in Caenorhabditis elegans], MAML-1 and p300 ⁄ CBP [38]. These complexes activate the transcription of the HES-1, -5, -7, HEY-1, -2, and HEYL genes encoding basic helix–loop–helix ⁄ orange domain transcriptional repressors [39]. Signal transduction from Notch ligands to the CSL–NICD–MAML-1 cascade is referred to as canonical Notch signaling pathway. In a non canonical pathway NICD can also interact with p50 or c-Rel in the nucleus to enhance nuclear factor (NF)-κB activity [38].

Unlike Notch2, Notch 1 and Jagged 1 are expressed abundantly in the proliferative zone located within the middle- third of the crypt in normal colon [40]. Jagged2 is expressed uniformly across the entire crypt. Several reports support the importance of Notch signaling for the intestinal progenitor compartment. Depletion of Hes-1 is associated with a significant increase in the secretory lineage of intestinal epithelial cells [41]. Conditional gut-specific inactivation of CSL leads to complete loss of proliferating crypt progenitor cells and their ultimate conversion into post-mitotic goblet cells [42]. Expression of NICD in the intestine inhibits differentiation of crypt progenitor cells thereby increasing undifferentiated transient amplifying cell evident from reciprocal gain-of-function studies [43].

Notch1 and Hes1 are significantly upregulated in colon adenocarcinomas [44], but remain normal in differentiated epithelial cells. Activation of Notch signaling is essential for the development of adenomas in ApcMin ⁄ + mice [42]. Hes1 is reported to suppress the expression of Kru¨ppel-like factor 4, a transcriptional repressor [45]. KLF4, the zinc finger-containing transcription factor, is highly expressed in terminally differentiated epithelial cells of the intestine [46], whose overexpression can inhibit colon cancer cell proliferation [47]. Haploinsufficiency of KLF4 augments the development of intestinal adenomas in ApcMin ⁄ + mice [48]. Both in adenomas and carcinomas expression of KLF4 is reduced relative to normal mucosa [49].

Role of notch signaling in colon cancer initiating cells (CCIC) or colon cancer stem cells has been investigated. NOTCH signaling is reported to be 10 to 30 fold higher in CCIC in comparison to commonly used colon cancer cell lines. Using small-molecule inhibitors and short hairpin RNA knockdown, it has been demonstrated that NOTCH prevents CCIC apoptosis through repression of cell cycle kinase inhibitor p27 and transcription factor ATOH1. NOTCH is also critical to intrinsic maintenance of CCIC self-renewal and the repression of secretory cell lineage differentiation genes such as MUC2 [50].

## Hedgehog signaling in colon cancer

The hedgehog signaling is named after the polypeptide ligand, an intercellular signaling molecule called Hedgehog (Hh) found in Drosophila. It is one of the key regulators of animal development and is present in all bilaterians [51]. The proliferation, migration, and differentiation of target cells are regulated by Hh signaling in a spatial, temporal, and concentration dependent manner [52].

In mammals, three Hedgehog homologues are present, of which Sonic hedgehog (Shh) is the best studied. Hh ligands binds to the transmembrane receptor Patched (Ptch) leading to the release of Ptch-mediated repression of Smo and consequent activation of the downstream signaling cascade that activates Gli family of transcription factors [52].

Emerging data support the existence of a pathologic Hh signaling happening in a similar manner which involves the tumor microenvironment [53]. Several reports from many human tumors have suggested that Hh signaling regulates cancer stem cells [54]. Hh signaling plays a critical role in the process of metastasis in solid tumors similar by regulating CSC functions [55].

HH-GLI is essential for the proliferation and survival of primary human colon carcinoma (CC) of all stages. HH-GLI is active in CC epithelial cells and affects both tumor growth and CD133+ cancer stem cells. Interestingly, It is reported that an increase in the levels of expression of HH-GLI signaling components in advanced and metastatic CCs, and their increased dependence on HH-GLI pathway activity, as compared with non-metastatic CCs [56].

## Concluding remark

**Figure 1:**
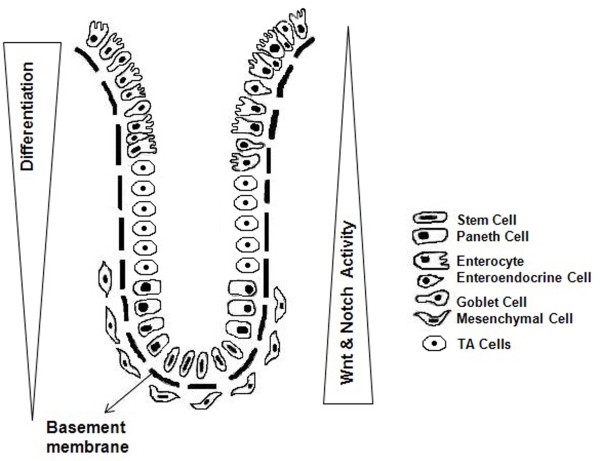
**Histology of coloic crypt (illustration adopted and modified: courtesy Anderson *et al *[**[57]**] & Simone *et al *[**[58]**]).**

Looking into the future, it is obvious that an important area of investigation will be colon CSCs and their signaling pathways. Understanding of the coordinated activity of these pathways might lead to more effective, early diagnosis of cancer and the development of therapeutic strategies for colorectal cancer, particularly the recurrence of the disease where CSCs play a prominent role (Figure 1).

## Competing interests

The authors declare that they have no competing interests.

## Authors’ contributions

RS carried out review of the literature, wrote the first draft and prepared the manuscript. MAPN, the principal investigator, was responsible for discussion, critical evaluation of the review and overall supervision of the final preparation of the manuscript. Both authors read and approved the final manuscript.
